# Cost effectiveness of lumbar epidural steroid injections

**DOI:** 10.1186/1748-7161-9-S1-P2

**Published:** 2014-12-04

**Authors:** Leah Carreon, Kelly Bratcher, Farah Ammous, Steven Glassman

**Affiliations:** 1 Norton Leatherman Spine Center, Louisville, Kentucky, USA

## Background

Economic value is an important component of healthcare policy decision making. The primary currency for comparing the value of competing healthcare interventions is the Cost per Quality Adjusted Life Year (QALY) gained.

## Aim

To determine cost/QALY gained for lumbar epidural steroid injections (LESI).

## Design

Longitudinal cohort.

## Methods

Patients who had an LESI between June 2012 and July 2013 and had EQ-5D scores available before and after LESIs were identified. Costs were calculated based on the Medicare Fee Schedule multiplied by the number of LESIs done between the two clinic visits. QALYs were calculated using the EQ-5D.

## Results

There were 257 patients (157 females, 100 males), mean age 60.0 years (21 to 88) years. Cost per LESI was $608, with most patients receiving 3 LESIs over one year (range 1 to 6). The mean QALY gained was -0.006. One hundred twenty-two patients (47%) had a gain in QALY (mean= 0.083) at a cost of $16,503 per QALY gained, 103 had a loss in QALY (mean = -0.112) and 32 had no gain in QALY. Ten of the 122 patients who improved, and 19 of the 135 patients that did not improve had medical comorbidities that precluded surgery. Of the 112 patients who improved without medical comorbidities, 31 (28%) subsequently had surgery. Of the 116 patients who did not improve and had no medical comorbidities, 45 (39%) subsequently had surgery. This proportion is statistically significantly different at p=0.05. Although the patients who had surgery even after improvement with LESI had a lower mean QALY gain (0.067) compared to those that did not have surgery (0.089), this was not statistically significant (p=0.260).

## Conclusions

This study indicates that at LESI may not be a cost-effective intervention in patients with low back pain. For the 122 patients who improved, cost per QALY gained was acceptable at $16,503. However for the 135 patients with no gain or a loss in QALY, the economics are unreportable with a cost per QALY gained being theoretically infinite. Further studies are needed in order to identify specific patient populations that will benefit from LESI, as the economic viability of LESI requires improved patient selection.

